# Testing the feasibility of the QuitAid smoking cessation intervention in a randomized factorial design in an independent, rural community pharmacy

**DOI:** 10.1186/s40814-024-01465-9

**Published:** 2024-02-26

**Authors:** Melissa A. Little, Taylor Reid, Matthew Moncrief, Wendy Cohn, Kara P. Wiseman, Candace H. Wood, Wen You, Roger T. Anderson, Rebecca A. Krukowski

**Affiliations:** 1 Department of Public Health Sciences, University of Virginia, School of Medicine, PO Box 800765, Charlottesville, VA 22908-0765 USA; 2 Cancer Control and Population Health, University of Virginia Cancer Center, Charlottesville, VA USA; 3 My Pharmacy, 2525 A Phillips Ave, Greensboro, NC USA

**Keywords:** Smoking cessation, Independent pharmacy, Rural health, Nicotine replacement therapy, Medication therapy management

## Abstract

**Background:**

Adult smoking rates in the USA are highest in economically depressed rural Appalachia. Pharmacist-delivered tobacco cessation support that incorporates medication therapy management (such as the QuitAid intervention) is a promising approach to address this need.

**Methods:**

Twenty-four adult smokers recruited between September and November 2021 through an independent pharmacy in rural Appalachia were randomized in a non-blinded 2 × 2 × 2 factorial design to (1) pharmacist delivered QuitAid intervention (yes vs. no); (2) combination nicotine replacement therapy (NRT) gum + NRT patch (vs. NRT patch); and/or (3) 8 weeks of NRT (vs. standard 4 weeks). Participants received 4 weeks of NRT patch in addition to the components to which they were assigned. Participants completed baseline and 3-month follow-up assessments. Primary outcomes were feasibility of recruitment and randomization, retention, treatment adherence, and fidelity.

**Results:**

Participants were recruited in 7 weeks primarily through a referral process, commonly referred to as ask-advise-connect (61%). Participants were on average 52.4 years old, 29.2% were male and the majority were white (91.6%) and Non-Hispanic (91.7%). There was a high level of adherence to the interventions, with 85% of QuitAid sessions completed, 83.3% of the patch used, and 54.5% of gum used. Participants reported a high level of satisfaction with the program, and there was a high level of retention (92%).

**Conclusions:**

This demonstration pilot randomized controlled study indicates that an ask-advise-connect model for connecting rural smokers to smoking cessation support and providing QuitAid for smoking cessation is feasible and acceptable among rural Appalachian smokers and independent pharmacists. Further investigation into the efficacy of a pharmacist-delivered approach for smoking cessation is needed.

**Trial registration:**

The trial was retrospectively registered at ClinicalTrials.gov. Trial #: NCT05649241.

## Key messages regarding feasibility

- What uncertainties existed regarding the feasibility?
  - ◦ It was unclear whether a pharmacist-delivered approach to smoking cessation is feasible in community pharmacies throughout rural Appalachia, where smokers have higher rates of tobacco use and are less likely to seek cessation support.
- What are the key feasibility findings?
  - ◦ This feasibility study indicates that the ask-advise-connect approach used by community pharmacists was successful in recruiting hard-to-reach smokers, and the QuitAid intervention was well received by both pharmacists and participants.
- What are the implications of the feasibility findings for the design of the main study?
  - ◦ This demonstration pilot randomized controlled study indicates that an ask-advise-connect model for connecting rural smokers to smoking cessation support and providing medication therapy management for smoking cessation is a promising study design to be tested in a full-scale trial among rural Appalachian smokers and independent pharmacists.

Adult smoking rates in the USA are highest in economically depressed rural Appalachia, specifically the Central and South Central subregions of Appalachia that include parts of Virginia, West Virginia, Tennessee, Kentucky, and North Carolina (henceforth referred to as “rural Appalachia”) [1, 2]. For example, 25–35% of residents report current smoking, far above the US national average of 14% [3, 4]. This region is also characterized by a high level of social vulnerability, which measures socioeconomic status, household composition and disability, minority status and language, and housing type and transportation. In a study comparing social vulnerability levels in Virginia, social vulnerability was on average 0.49 in Virginia compared to 0.63 in rural areas and 0.58 in rural Appalachian counties (range =0–1, with higher values indicating more vulnerability), and social vulnerability was directly correlated with smoking rates [5]. Smokers living in rural Appalachia are more likely to start smoking at a younger age, smoke daily and in excess of 15 cigarettes per day, and have poorer cessation-related outcomes [6, 7]. Additionally, smokers in rural Appalachia face financial barriers for pharmacotherapy, exacerbated by the fact that they often have limited awareness of available smoking cessation programs and resources [8]. Thus, there is a need to increase the reach of available smoking cessation programs to assist rural smokers to make a successful quit attempt [9].

One key smoking cessation strategy is nicotine replacement therapy (NRT)—an effective, safe, and easily accessible resource [10]. In our survey of 49 independently owned community pharmacies in rural Appalachia, 100% reported selling NRT [11]. Despite the fact that NRT increases the rate of quitting by 50–60% [10], it is often not adhered to in terms of dose and duration [10, 12, 13]. In a systematic review of barriers and facilitators to NRT adherence, the most important factors associated with adherence were related to reflective motivation, such as perceptions about NRT and quitting, physical capability, namely level of nicotine dependence and withdrawal symptoms, and automatic motivation, such as stress, depression and alcohol use, consistent with the capability, opportunity, motivation, and behavior (COM-B) model [14]. Similarly, population-based studies have found that over a third of smokers believe pharmacotherapy would be of little help and may even reduce their chances of successfully quitting [15]. However, the evidence suggests that combination NRT (fast-acting form [gum] + patch) further increases cessation (RR 1.3; 95% CI 1.2–1.4; 14 trials) although the optimal duration is unclear [16]. The lack of evidence on the optimal duration is problematic in terms of informing public policy related to NRT; for example, there is not a standard amount of free NRT provided by state quitlines; 39% provide 2 weeks, 16% provide 4 weeks, 29% provide 8 weeks, and 16% provide another amount [13].

Given that higher levels of NRT adherence are associated with cessation, it is critical that interventions for rural smokers promote adherence to NRT [17]. A meta-analysis found that medication adherence interventions led to a small improvement in adherence and long-term smoking cessation rates (RR 1.16, 95% CI 0.96 to 1.40) [18], but the available evidence was of low quality. Therefore, interventions are needed to improve suboptimal NRT adherence rates [18]. One possible avenue for disseminating NRT adherence interventions to rural communities is through independent pharmacies, given their clinical expertise, repeated patient interactions, and centralized placement in the community.

In the USA, 91% of Americans live within 5 miles of an independent pharmacy [19–21]. However, to date, independent pharmacies have been under-utilized in the provision of tobacco cessation [22], with only 14% of independent pharmacists in the USA reporting providing smoking cessation services [23]. Given the broad availability of smoking cessation medications in pharmacies and the fact that 17 states currently allow pharmacists to prescribe smoking cessation medications [24], an opportunity exists to train pharmacists to become a recognized local resource for smoking cessation [20]. Additionally, we surveyed independent pharmacists in rural Appalachia and found that 90.3% were interested in providing smoking cessation resources and treatment [11]. A recent meta-analysis of pharmacy-assisted smoking cessation interventions found that interventions were effective, but the evidence was low quality [25]. Thus, the optimal pharmacist-led intervention for smoking cessation remains unclear [25].

Furthermore, barriers to pharmacist delivered smoking cessation support need to be addressed [26]. For instance, only 11.4% of independent pharmacists who responded to our survey felt that they had a “great deal” of experience with providing smoking cessation resources [11]. Additionally, pharmacists are not currently compensated for providing smoking cessation services. As such, pharmacists tend to curb these activities in lieu of maximizing their medication dispensation for which there is clear reimbursement.

Medication therapy management (MTM) may be a strategy to overcome financial barriers for pharmacist-delivered smoking cessation support. MTM services, which have been used to manage chronic diseases such as diabetes, generally consist of medication review, an individual medication record and medication-related action plan, intervention and/or referral, documentation, and follow-up [27]. The Medicare Prescription Drug, Improvement, and Modernization Act paved the way in 2013 for pharmacists to receive compensation for providing medication expertise [28, 29]. Recently, MTM services have been widely adopted due to standard documentation and billing systems [28]. Research has found that independent pharmacies that deliver MTM lead to positive clinical outcomes for patients [28, 30–32] and are well received [33]. While MTM has not been used for the provision of smoking cessation, these findings suggest that a MTM-based approach to smoking cessation that utilizes a standard documentation and billing platform could provide a highly disseminable avenue for pharmacist-delivered smoking cessation support for hard-to-reach rural smokers.

To determine the feasibility of a pharmacist-delivered MTM-based approach to smoking cessation in rural Appalachia, we conducted a factorial randomized controlled trial. A factorial design was chosen to determine the feasibility of conducting a factorial experiment in independent pharmacies with rural Appalachian smokers. We also sought to determine feasibility of recruitment and randomization, retention, treatment adherence, and treatment fidelity of rural smokers through a single independently owned pharmacy in the region.

## Methods

The current study used a 2 × 2 × 2 factorial design to implement three tobacco treatments with 24 rural smokers in Appalachia: (1) pharmacist delivered novel MTM intervention, QuitAid (yes vs. no); (2) combination NRT gum + NRT patch (vs. NRT patch alone); and/or (3) 8 weeks of NRT (vs. standard 4 weeks). All participants received at least 4 weeks of NRT patch in addition to any of the other components they were assigned to receive. These components were chosen for their ability to be easily disseminated in a rural setting.

### Participants and procedures

Between September and November 2021, we recruited 24 adult smokers through their local independent pharmacy in rural Appalachia. Participants were primarily recruited through an ask-advise-connect model in which pharmacists asked potential participants if they were smoking, advised them to quit, and connected them to our study if they were interested [34]. Other recruitment methods included flyers on prescription bags and store signage. Adult smokers interested in participating in the study were then screened telephonically by research staff to determine eligibility. To be eligible, smokers must have smoked at least 5 cigarettes per day for the past 6 months and be willing to set a quit date in the next 30 days, own a cell phone, be over 18 years of age, not be pregnant or planning to become pregnant in the next 6 months, and not have any medical contraindications to using NRT. If eligible, participants were consented and provided an opportunity to ask questions. Participants signed their consent form through a mailed paper consent form that they signed and returned to the study staff in a pre-stamped envelope, electronically through DocuSign, or were given the option to sign a paper copy at their local pharmacy. Individuals who were not eligible or did not wish to participate were provided with alternative smoking cessation resources (e.g., referral to the state quitline). To account for the low literacy levels of the target population, participants were given the option to have the consent form and all assessments read to them either over the phone by a member of the study team, or for the assessments, they could also go into the pharmacy and have someone blinded to their treatment condition read them the survey. Consented participants then completed the baseline assessment and were randomized to one of the 8 treatment combinations (see Table 1). Equal allocation across treatment combinations was used and balance between treatment combinations was maintained by use of randomly permutated blocks with unequal block sizes of 8 and 16 generated by the study biostatistician; study staff were blinded to assignment until it was revealed by the study database. This study was approved by the Health Sciences Research Institutional Review Board at the University of Virginia.

**Table 1:** Randomized conditions

Condition	QuitAid intervention	NRT duration	NRT product
1	Yes	8 weeks of NRT	NRT patch + NRT gum
2	Yes	8 weeks of NRT	NRT patch
3	Yes	4 weeks of NRT	NRT patch + NRT gum
4	Yes	4 weeks of NRT	NRT patch
5	No	8 weeks of NRT	NRT patch + NRT gum
6	No	8 weeks of NRT	NRT patch
7	No	4 weeks of NRT	NRT patch + NRT gum
8	No	4 weeks of NRT	NRT patch

3 participants were randomized to each treatment condition

### Treatment components

#### Nicotine replacement therapy

NRT was distributed by the pharmacy through a standing physician order which allowed the pharmacists to bill the participant’s insurance for the cost of NRT. The participant’s insurance was billed first, and the study paid any remaining balance from co-insurance or co-pays. For participants who did not have insurance, or their insurance did not cover the NRT, the study covered the entire cost of the medication. Participants had the option to pick up their NRT at the pharmacy or have the pharmacy mail the amount of NRT they were randomized to receive (4- or 8-week supply, plus patches only or patches plus gum). NRT was distributed in 4-week increments to align with insurance coverage (many insurance plans only cover for 4-weeks of NRT at one time). The first 4-week supply of NRT was filled within 7 days of enrollment. For participants randomized to receive an 8-week supply of NRT, the second 4-week supply was distributed during week 3 to ensure that the participant did not run out. NRT shipments included standard NRT use guidelines with details on proper use and potential side effects.

#### QuitAid intervention

The QuitAid MTM intervention addressed perceptions (e.g., motivation, self-efficacy, beliefs) and practicalities of using NRT (e.g., monitoring NRT use, providing reminders) that can be mapped onto the capability, opportunity, motivation, and behavior (COM-B) model [35]. The intervention targets include (1) goals and planning (e.g., strategies to remember to use NRT), (2) shaping knowledge (e.g., instructions on how to properly use NRT), (3) natural consequences (e.g., importance of continuing to use NRT throughout the entirety of the quit attempt), (4) associations (e.g., strategies to remember to use NRT), (5) comparison of outcomes (e.g., decisional balance about using NRT), (6) reward and threat (e.g., identifying self-rewards for achieving goals related to using NRT), (7) regulation (e.g., encouraging adherence to NRT), (8) antecedents (e.g., avoiding exposure to cues to smoke), and (9) self-belief (e.g., focusing on past successful behavior change attempts). These targets are in line with a systematic review of barriers and facilitators to adherence to NRT [14]. These strategies have previously been used to effectively promote cessation in clinical trials [36–39]. The QuitAid treatment included 1 in-person coaching session with the participant’s local pharmacist, and 5 follow-up telephonic coaching sessions. The first session included a discussion to address any negative beliefs about the use of NRT while strengthening the participant’s motivation and commitment to using NRT [18]. Participants were also instructed on proper use of the NRT they were randomized to receive (patches only or gum plus patches), useful tips to avoid common problems with NRT (e.g., skin irritation, cravings, use patch plus gum in combination), and strategies that have previously been used to increase adherence to NRT [18, 40]. Participants were instructed to begin using the NRT medication immediately, even if they were still smoking. Within 2 days of the first session, participants received a call from their pharmacist or technician to address any questions or concerns they may have had about their NRT use. The remaining weekly proactive check-in calls occurred 7, 14, 21, and 28 days from their initial call. During these weekly calls, the pharmacists addressed any negative beliefs about NRT, monitored the use of NRT, provided feedback regarding NRT use, and provided additional support to overcome any barriers to adherence [18]. All sessions occurred within 4 weeks from the time of enrollment.

### Pharmacist training

Prior to the start of the study, pharmacists and technicians participated in two virtual trainings led by research staff to introduce key concepts and skills required by the program, build self-efficacy and comfort with the approach, and generate enthusiasm and commitment to the program. The trainings presented an overview of the theoretical underpinnings and evidence-base for the intervention, as well as detailed instruction about the ask-advise-connect method for identifying and recruiting smokers into the study and delivering the QuitAid intervention. They also completed a 1-h BMJ learning course on motivational interviewing in brief consultations [41]. Lastly, given their access to participant data, all pharmacists and technicians were required to complete online human subjects training [42].

### Program implementation

The pharmacy had a dedicated on-site implementation leader to oversee the daily operations of the study and provide on-going support to other pharmacists or technicians related to study activities. The implementation leader worked directly with the study team to ensure open communication and fidelity to the treatment protocol and received a monthly stipend during the phases of active recruitment and implementation. Additionally, the pharmacy was reimbursed for providing the QuitAid MTM sessions, similar to what they would be reimbursed by an insurance company for providing MTM interventions for other chronic diseases, such as chronic heart failure, hypertension, and diabetes. We reimbursed pharmacies $40 for the initial in person visit and $20 for each additional visit (total of $140 per patient if they completed all 6 sessions).

### Measures

#### Participant measures

The baseline and 3-month follow-up assessments were self-administered via either a secured web-based platform or mailed paper copies. The baseline survey assessed demographics (e.g., gender, age, ethnicity, marital status, race, education), pharmacist provider trust (11 items; *α* = 0.89; 1 = strongly disagree to 5 = strongly agree) [43], and previous behavioral smoking cessation aids used in the past 12 months (i.e., quitline, one-on-one in person counseling, class or support group, web-based program, smartphone application, and text-messaging program). At baseline and 3-month follow-up, tobacco use, quit attempts, and nicotine dependence using the Fagerström Test for Nicotine Dependence (FTND) were assessed [44]. The FTND includes six items that measure cigarette consumption, compulsion to use, and dependence. Items are summed to create a total score of 0–10 with higher scores indicating more intense physical dependence on nicotine.

##### Primary outcome measures (measures of feasibility)

Process measures were assessed through screening logs and participant surveys. Pace of recruitment was measured by (a) the proportion of smokers recruited from each source (e.g., ask-advise-connect, posters, prescription bag advertisements) and (b) the number recruited per month. We defined recruitment success if we were able to recruit eight smokers per month over the course of 3 months from a single pharmacy, which we anticipate would be sufficient to conduct a larger study within the typical budget and time period. Feasibility of randomization was determined by the number of smokers that were approached and screened in order to randomize 24 smokers (e.g., ineligible smokers, smokers that do not consent) [45, 46]. We hypothesized a 25% screen failure rate and 5% dropout/withdrawal rate. Retention was assessed by the proportion of smokers who completed the 3-month follow-up [47]. We defined retention success as more than 80% of the sample was retained at the 3-month follow-up. Receipt of the QuitAid intervention was defined as the number of QuitAid sessions the participant received [47]. Receipt of NRT was defined as the percent of NRT used and was collected from participants at the 3-month follow-up [18, 47]. We defined dose receipt of the interventions success as more than 75% of the intervention components were delivered. At the 3-month follow-up, satisfaction with treatment components were assessed (e.g., “The program kept my interest and attention”; see Table 3) [47]. Items were assessed on a 6-point Likert scale with 1 = strongly disagree to 6 = strongly agree [48, 49]. We defined satisfaction with treatment components success as mean scores greater than or equal to 4.

##### Secondary outcomes (tobacco use)

Tobacco use outcomes to examine overall changes in tobacco use were collected. Biochemically verified self-reported point prevalence tobacco abstinence at the 3-month follow-up was collected [50]. If a participant reported no tobacco use in the previous 7 days at the 3-month follow-up, they were asked to visit their local pharmacy to provide a saliva sample within 48 hours to biochemically verify their abstinence. Previous research has determined that optimal serum cotinine concentrations for discriminating tobacco abstinence were 3.08 ng/mL with a high degree of sensitivity and specificity (> 96%) [51]. Thus, in the current study, a concentration of≥ 3 ng/mL cotinine was set as the cutpoint for those who were smoking.

#### Pharmacist measures

Pharmacists and technicians completed a baseline survey related to their demographic (i.e., age, gender, and education) and professional characteristics (i.e., years of experience as a pharmacist, and experience providing tobacco cessation resources) via a secured web-based platform. Following completion of the factorial experiment, pharmacists and technicians completed a follow-up survey to assess confidence in the approach (e.g., “How confident are you that you answered participants’ questions about NRT use adequately?” 0 = not at all to 10 = extremely confident), perceived efficacy in the program (e.g., “How successful do you think smoking cessation MTM will be in reducing tobacco use among your customers?” 1 = not at all successful to 10 = extremely successful), and fidelity to the program (e.g., “Overall, how much did you adhere to the lesson plans (deliver them as written)?” 0 = not at all to 10 = extremely close).

### Sample size

**Fig. 1:**
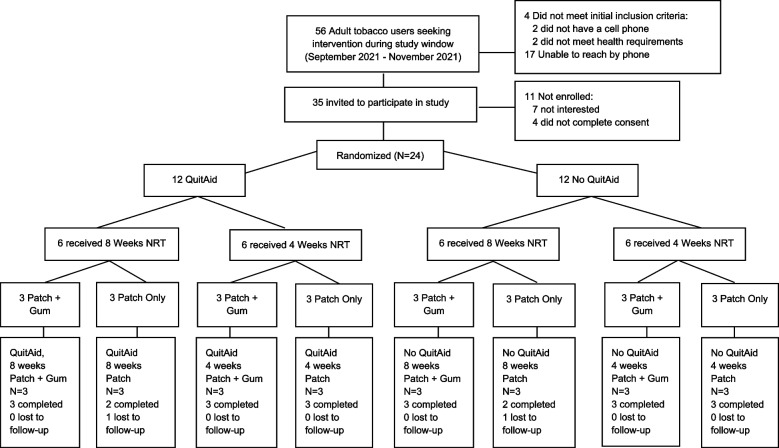
CONSORT diagram. QuitAid MTM (medication therapy management)

We chose a sample size of 3 per group based on the recommendations outlined in Julious [52], which recommends 12 participants per treatment for use in pilot feasibility trials based on the rationale about feasibility, the precision regarding the mean and variance, and regulatory considerations [52, 53]. Although the current study was a factorial design with 8 conditions, every treatment was delivered to half the sample, or 12 participants per treatment. Thus, while only 3 participants were randomized to each of the 8 conditions, 12 participants received each of the treatment components, in accordance with the recommendations by Julious (see Fig. 1) [52].

### Data analysis

Descriptive statistics were used to summarize demographics, feasibility of recruitment, feasibility of randomization, retention, fidelity of implementation, program satisfaction, and overall changes in tobacco use behaviors. Statistical analyses were conducted in SPSS version 9.0 (SPSS Inc, Chicago, IL).

## Results

### Feasibility of recruitment, randomization and retention

We successfully recruited all 24 smokers in 7 weeks (approximately 3 smokers per week) from a single pharmacy. The majority were recruited using ask-advise-connect (61%), while the rest were self-referred from in store posters (18%), prescription bag advertisements (6%), and word of mouth (15%). Of the 56 smokers referred to the study, 39 (69.6%) were interested in being screened for eligibility (see Fig. 1). Following screening, 4 (10.3%) were ineligible and 7 (17.9%) were no longer interested in participating. Among the 28 eligible individuals, 85.7% were enrolled. There was a high level of retention, with 22 (92%) of the participants completing the 3-month follow-up assessment.

As shown in Table 2, participants were on average 52.4 years old, 29.2% (*n* = 7) were male and the majority were white (*n* = 22, 91.6%) and Non-Hispanic (*n* = 22, 91.7%). A third of the sample was married (*n* = 8), and only 20.8% (*n* = 5) were currently employed. Less than half (*n* = 9, 39.1%) had some college education, 62.5% (*n* = 15) reported less than $30,000 in annual household income, and the majority had some form of health insurance coverage (*n* = *21,* 87.5%). All participants reported smoking at least 100 cigarettes in their lifetime. The average age at initiation was 16, the average FTND score was 5.2 (*standard deviation [SD]* = 2.3), and the majority of participants reported smoking everyday (*n* = 22, 91.7%). Roughly 45% (*n* = 13) of participants reported trying to quit in the previous 12 months, but none reported using a behavioral-based smoking cessation aid (not shown in the table). Finally, participants reported a high level of trust in local independent pharmacists (*mean* = 3.9, *SD* = 0.6).

**Table 2:** Baseline characteristics of participants (*N* = 24)

	Mean (standard deviation)/*N* (%)
Age, mean (SD)	52.4 (11.2)
Male, *N* (%)	7 (29.2%)
Race, *N* (%)	
White	22 (91.6%)
Other	2 (8.4%)
Hispanic/Latino ethnicity, *N* (%)	2 (8.3%)
Marital status, *N* (%)	
Married or living as married	8 (33.3%)
Widowed	3 (12.5%)
Divorced/separated	6 (25%)
Single, never married	6 (25%)
Occupational status, *N* (%)	
Employed	5 (20.8%)
Unemployed	3 (12.5%)
Homemaker	2 (8.3%)
Retired	4 (16.7%)
Disabled	9 (37.5%)
Household income, *N* (%)	
$10,000 or less	5 (20.8%)
$10,001–$20,000	6 (25%)
$20,001–$30,000	4 (16.7%)
Over $30,000	7 (29.2%)
Education, *N* (%)	
Less than high school	6 (26.1%)
High school graduate or GED	8 (34.8%)
Some college	9 (39.1%)
Health insurance coverage, *N* (%)	
Employer sponsored insurance	4 (16.7%)
Medicare, Medicaid, or medical assistance	16 (66.7%)
Private	1 (4.2%)
Uninsured	2 (8.3%)
Did not disclose	1 (4.2%)
Smoked 100 cigarettes in lifetime, *N* (%)	24 (100%)
Age at smoking initiation, mean (SD)	16 (7)
Current smoking status, *N* (%)	
Every day	22 (91.7%)
Some days	2 (8.3%)
Past 12 month quit attempts, *N* (%)	
I have never tried to quit in the past 12 months	11 (45.8%)
Once	4 (16.7%)
2–3 times	5 (20.8%)
4 or more times	4 (16.7%)
Fagerstrom test for nicotine dependence, ^a^ mean (SD)	5.2 (2.3)
Pharmacist trust, ^b^ mean (SD)	3.9 (0.6)

*SD* Standard deviation

^a^10-point scale with higher scores indicating greater nicotine dependence

^b^5-point Likert scale with 1 = strongly disagree to 5 = strongly agree

### Dose of the interventions

There was a high level of adherence to the interventions, with 85% (*n* = 61) of MTM sessions completed, and 83.3% (*n* = 15) reporting more than 75% of the patch used and 54.5% (*n* = 6) reporting more than 75% of gum used.

### Program satisfaction

Overall, there was a high level of satisfaction with the program (see Table 3), with a mean score for the overall program of ≥ 4.2. The most strongly endorsed item was the belief that the program helped them quit or cut down their cigarette use (*mean* = 4.7, *SD* = 1.0). Participants also felt that the NRT patch was helpful and easy to use ≥ 4.6. Finally, participants reported satisfaction with the QuitAid component.

**Table 3:** Summary of intervention satisfaction by participants (*N* = 22)

	Mean (standard deviation)
**Overall program satisfaction**	
I am satisfied with the program in general	4.4 (1.0)
I like the way the program looked	4.5 (1.1)
The program kept my interest and attention	4.3 (1.2)
The program fit me	4.2 (1.1)
This program was more helpful than other smoking cessation programs I’ve tried in the past	4.3 (1.4)
This program helped me quit or cut down my cigarette use	4.7 (1.0)
I would recommend this program to a friend or family member who was interested in quitting smoking	4.6 (1.0)
**Satisfaction with the NRT patch**	
The NRT patch was helpful	4.6 (1.0)
I understood how to use the NRT patch	4.6 (1.0)
The program made it easy for me to use NRT patch	4.6 (1.0)
NRT patch is helpful for smokers trying to quit	4.4 (1.0)
NRT patch is a hassle to use	1.5 (0.9)
**Satisfaction with the QuitAid intervention**	
My pharmacist was knowledgeable about the use of NRT and side effects	4.1 (1.4)
I felt comfortable talking to my pharmacist about my quit attempt	4.7 (1.3)
Finding time to meet with a pharmacist is difficult	1.5 (1.2)
There were too many sessions with the pharmacist	1.2 (0.4)

Items were assessed on a 6-point Likert scale with 1 = strongly disagree to 6 = strongly agree

### Secondary outcomes

At the 3-month follow-up, 31.8% (*n* = 7) of participants reported abstinence and 88.9% (*n* = 16) reported attempting to quit during the study. Additionally, there was a decrease in the FTND between baseline and follow-up (*mean* = −1.56, *SD* = 1.7) (Table 4).

**Table 4:** Secondary tobacco use outcomes (*N* = 22)

	M (standard deviation) or *N* (%)
Change in FTND, ^a^ M(SD)	−1.56 (1.7)
Point prevalence abstinence, ^b^* N* (%)	7 (31.8%)
Quit attempts	16 (88.9%)
NRT adherence	
75% of patch provided, *N* (%)	15 (83.3%)
75% of gum provided, *N* (%)	6 (54.5%)

*SD* Standard deviation, *FTND* Fagerstrom Test for Nicotine Dependence

^a^10-point scale with higher scores indicating greater nicotine dependence

^b^Point prevalence abstinence using an intent to treat assumption

### Pharmacist measures

Participating pharmacists were on average 39.7 years old (*SD* = 1.53), with 15.7 years of experience working in a pharmacy (*SD* = 5.51). Two (66.7%) were female, all (*n* = 3) had received a PharmD. They reported “a little” previous experience with providing tobacco cessation resources to customers (*mean* = 2.67, *SD* = 0.58). Overall, pharmacists felt confident in their delivery of the program (*mean* = 7.5, *SD* = 0.71), had a high level of enthusiasm towards the program (*mean* = 9, *SD* = 1.41), and perceived the program to be effective (*mean* = 8, *SD* = 1.41; see Table 5). They also reported a high degree of comfort with the approach (*mean* = 7.5, *SD* = 0.71), adherence to the program guides (*mean* = 8, *SD* = 0), and ease of delivery (*mean* = 8, *SD* = 0).

**Table 5:** Pharmacist and technician perceptions of the program (*N* = 3)

	Mean	Standard deviation
How confident are you that you…		
Did a good job delivering smoking cessation MTM	7.5	0.71
Answered participants’ questions about NRT use adequately	8.5	2.12
Got participants to engage in discussions about NRT use	9	0
Liked the program	9.5	0.71
Perceived participant engagement in the program	8	1.41
Perceived efficacy of the program	8	1.41
Enthusiasm towards the program	9	1.41
Comfort with the approach	7.5	0.71
Adherence to the program guides	8	0
Ease of delivery	8	0

10-point scale with high scores indicating more agreement

## Discussion

We demonstrated the feasibility of delivering an MTM smoking cessation intervention, QuitAid, and conducting a randomized controlled trial with a 2 × 2 × 2 factorial design in an independent pharmacy in rural Appalachia. We successfully recruited participants and retained 92% of them at the 3-month follow-up. We accomplished our randomization goal with a complex randomization scheme and provided the appropriate intervention components without cross-contamination. Overall, the intervention was well received by participants and pharmacists, with high satisfaction ratings across the treatment components and high level of adherence to the intervention components. In addition, participants found their independent pharmacists to be knowledgeable and approachable. The program helped participants cut down and quit, with most participants setting a quit date. Finally, 31.8% of participants reported abstinence at the follow-up, which is a typical quit rate in a combined behavioral and pharmacological interventions in both pharmacy [54] and non-pharmacy settings [10, 55–57]. In the pharmacy setting, 7-day point prevalence abstinence rates at a 3-month follow-up ranged from 20.7 to 27.5% [54], which is similar to the findings in the current study.

There was a high level of adherence to NRT in the current study, particularly the NRT patch. While there was a lower utilization rate of the NRT gum, given that this was an adjunct treatment to the NRT patch, and participants who received the gum were told to use it when they had a breakthrough craving, one would expect this rate to be lower [15]. Previous research has found individual differences in the preference for various forms of NRT products [58]. Comparing the NRT gum, patch, nasal spray, and inhaler, the NRT gum was the least popular option among smokers attempting to quit, while the NRT patch was the most popular, and these findings were consistent across both men and women, although the difference was more marked for women [58]. Interestingly, prior experience using the NRT product had no effect on preference for the patch, but did affect preference for the gum, with individuals who had prior experience using the gum less likely to prefer that form of NRT [58]. This could be because the name “gum” misleads users to think that they chew the NRT gum like traditional gum, when in fact it is mean to be activated by biting down on it until one feels a tingling sensation, and then “parked” between the inside of one’s cheek and gums [59]. This process is repeated until the tingling stops, usually after about 30 min of use. Thus, individuals may not be using it correctly, or may find the gum to be strange due to their expectations about the product. Given the importance of proper utilization of NRT, both in terms of dose and duration, understanding individual preferences for NRT and the effect on utilization should be considered when designing smoking cessation trials.

Participants were very positive about their independent pharmacists and reported a high level of trust. We hypothesize that it was this trust that allowed us to reach our recruitment goal in 3 months primarily utilizing an ask-advise-connect method and could also explain the high level of program satisfaction. An ask-advise-connect model has been adopted nationally to promote utilization of publicly available smoking cessation programs. In 2019, there were over 200,000 referrals from physicians and pharmacists connecting smokers to state quitlines [13]. In our previous research with independent pharmacists in rural Appalachia, an ask-advise-connect model was well received; 83.7% were comfortable asking a customer about their tobacco use, 90.7% were confident they could advise a customer on the use of NRT, and 93.3% felt that it was feasible to connect tobacco users to a state quitline [60]. In the current study, pharmacists reported a high level of confidence in the approach and perceived participant engagement. In general, the pharmacists felt that they implemented the program as written and believed it was effective in reducing tobacco use among their participants. Thus, the current study suggests that this method is also effective at recruiting potentially hard to reach rural Appalachian smokers. Interestingly, all participants who signed up for the current study reported not having sought any behavioral smoking cessation support in the 12 months before enrolling. Thus, an ask-advise-connect model to recruit rural Appalachian smokers out of independent pharmacies shows great promise for engaging this population.

While it appears that pharmacists had the necessary time to recruit participants and deliver the QuitAid intervention, several adaptions were made at the onset of the study to ensure project success. Given that the pharmacy had several roles in this study (i.e., recruitment, NRT distribution, QuitAid implementation, biochemical verification of abstinence) in addition to routine pharmacy activities (e.g., medication dispensing, vaccinations, COVID testing, and MTM delivery for other chronic conditions), we recognized the need for an on-site implementation leader. This individual needed to be located within the pharmacy (pharmacist or technician) to oversee the daily operations of the study and provide on-going support to other pharmacists or technicians to achieve a high level of fidelity to study-related activities. Given that this was a research study with additional documentation and monitoring requirements, it is unclear if this individual would be needed in a real-world implementation of the program.

We also adapted our initial plans for training pharmacists and technicians. We had originally planned to hold a 1-day in-person training prior to launching the study. However, based on feedback from our independent pharmacists and the ongoing COVID-19 pandemic, we revised our training plans to provide self-paced web-based training and two virtual coaching sessions. In the future, it will be important to incentivize pharmacists to receive this training. One option is to provide CME credits for completing tobacco treatment specialist training and training on motivational interviewing. There are also freely available self-paced comprehensive tobacco cessation trainings for pharmacists through the Rx for Change: Clinician-Assisted Tobacco Cessation [61]. These modules cover ask-advise-connect, prescribing tobacco cessation medication, behavioral counseling, and pharmacotherapy, and could be successfully utilized in implementation trials to train large numbers of pharmacists and technicians.

There were several weaknesses of the current study that should be considered when interpreting the results. First, the study was intentionally small, recruiting 24 smokers from 1 independent pharmacy to determine feasibility of the approach. Therefore, future fully powered studies are necessary to determine efficacy of both the approach (e.g., connecting rural smokers to tobacco cessation services) as well as the QuitAid intervention. Additionally, because we only had 3 participating pharmacists and 1 pharmacy technician involved in the study, the results related to pharmacists’ and technician’s perceptions of the program are very imprecise and so interpretation is limited. As a result, it is unclear whether the current approach and intervention would be feasible in other independent pharmacies. For example, we had a high level of QuitAid session delivery (82% of sessions were delivered). It is possible in a larger trial with pharmacists with more diverse workflows and demands on their time that such a high level of implementation might not occur. However, the current study was conducted during COVID-19, when the pharmacy was overtaxed with conducting COVID-19 tests and administering COVID-19 vaccines. Given that this pharmacy was still able to achieve a high level of implementation during a very challenging time is promising. To ensure similarly high rates of implementation in the future, all pharmacists and technicians within each pharmacy should be trained in the ask-advise-connect method and QuitAid intervention delivery to provide coverage whenever the pharmacy is open. Additionally, it is possible given the association between cost of smoking cessation medication and adherence that participants without insurance may have had higher adherence rates. However, because of the small sample size, it was beyond the scope of this study to explore this relationship further. Future studies should consider examining whether there is differential adherence based on health insurance coverage, and thus differential quit rates.

Most of our sample (70.8%) were female, despite the fact that the limited epidemiologic data on smoking prevalence among rural Appalachian residents suggests that men are more likely to smoke compared to women [62, 63]. However, previous research has found that women are more likely to utilize recommended cessation resources, such as calling a quitline or using the nicotine patch [64]. Thus, it is unclear whether the current study is appealing to male smokers, and whether the current methods are feasible for recruiting balanced samples in terms of sex. Future studies should determine whether other modalities increase recruitment of male smokers into clinical trials.

Finally, given the nature of the current study to determine the feasibility of the approach, we were not powered to detect differences between treatment components. Therefore, future studies should explore whether these treatments increase cessation among this population.

This pilot randomized controlled study indicates that an ask-advice-connect model for connecting rural smokers to smoking cessation support and providing MTM for smoking cessation is feasible and acceptable among rural Appalachian smokers and independent pharmacists. The ask-advice-connect model was successful in recruiting hard-to-reach smokers, and the QuitAid intervention was well received by both pharmacists and participants. Therefore, an MTM-based approach for smoking cessation delivered by independent pharmacists could provide a ready and highly disseminable avenue for smoking cessation support for hard-to-reach rural smokers.

## Data Availability

The data from the current study is not publicly available due to institutional review board requirements but can be made available from the corresponding author on reasonable request.
